# Biosensors for Antioxidants Detection: Trends and Perspectives

**DOI:** 10.3390/bios10090112

**Published:** 2020-09-01

**Authors:** Melinda David, Monica Florescu, Camelia Bala

**Affiliations:** 1 Department of Fundamental, Prophylactic and Clinical Disciplines, Faculty of Medicine, Transilvania University of Brasov, Str. Universitatii no. 1, 500068 Brasov, Romania; melinda.david@unitbv.ro (M.D.); florescum@unitbv.ro (M.F.); 2 Laboratory for Quality Control and Process Monitoring, University of Bucharest, 4-12 Elisabeta Blvd., 030018 Bucharest, Romania; 3 Department of Analytical Chemistry, University of Bucharest, 4-12 Elisabeta Blvd., 030018 Bucharest, Romania

**Keywords:** antioxidants, biosensor, nanomaterials

## Abstract

Herein we review the recent advances in biosensors for antioxidants detection underlying principles particularly emphasizing advantages along with limitations regarding the ability to discriminate between the specific antioxidant or total content. Recent advances in both direct detection of antioxidants, but also on indirect detection, measuring the induced damage on DNA-based biosensors are critically analysed. Additionally, latest developments on (bio)electronic tongues are also presented.

## 1. Introduction

Antioxidants (AOx) play an important role, since they represent a defense system of all aerobe organisms, especially in the case of humans, where as a consequence of the metabolic and physiological processes, unstable reactive substances are generated as byproducts [1]. These unstable substances are referred to as reactive oxygen/nitrogen species (ROS/RNS), and they are molecules containing oxygen or nitrogen, with one or more unpaired electrons, making them very reactive. Some of the most important oxygen-derived molecules are the radicals of hydroxyl (-OH), singlet oxygen (O_2_) and superoxide anion (O_2_^-^) and hydrogen peroxide (H_2_O_2_) as non-radical. Nitrogen-derived species are nitric oxide (NO), peroxynitrite (HNO_3_^-^) or dinitrogen dioxide (N_2_O_2_) [2]. An increase of reactive oxygen species can overwhelm the natural antioxidant system of the organism leading to oxidative stress (OS). An increasing number of medical studies correlate the presence of OS to various disorders and medical conditions caused by damage inflicted to healthy cells [3]. For a variety of cardiovascular diseases, including strokes, OS has been, at least partially, viewed as one common etiology, with an increased ROS production of the organism [4]. An abnormal oxidation status has further been linked with chronic diseases such as diabetes [5] and neurological diseases such as Alzheimer’s [6,7]. In the case of cancer, it has already been proven that the level of ROS is increased [8,9].

Antioxidants have the role of reducing the adverse effects caused by ROS or RNS, and can herewith be divided in two categories. The first category consists of primary, or so-called chain-breaking AOx (e.g., vitamins, carotenes, phenols), which inhibit the oxidation of biomolecules; the second category comprises AOx that prevent ROS/RNS formation. The essential characteristic of an AOx is its property to donate the hydrogen from its active hydroxyl group (A-OH) in order to generate more stable radicals [10]. In more detail, AOx use two major mechanisms to deactivate radicals: hydrogen atom transfer (HAT), mentioned above, and single electron transfer (SET). Through the SET mechanism, the AOx transfers one electron (A-OH^+^) in order to reduce any compound, being followed by deprotonation in solution [11]. Herewith, the distinction between AOx and phenolic compounds is also a question of oxidation potential, as thoroughly described by Buratti et al. [12]. The antioxidant capacity of phenols depends on their redox properties, which depend on the presence of a phenolic aromatic ring with hydroxyl substituents in their chemical structure. It is well known that anodic peaks at low oxidation potentials (below ~ 0.5 V) occur for compounds with significant antioxidant activity [13], while, at potentials greater than +0.6 V, all phenolic compounds will be oxidized [12]. Some major benefits of AOx are described in the literature. Vitamin C reduces the incidence of degenerative and chronic diseases [14], while polyphenols prevent cardiovascular diseases and show anti-viral and anti-inflammatory properties [15]. Since many fruits, vegetables and medicinal herbs are known for their high content of AOx, proper alimentation and food supplements are often advised. The antioxidant properties of different plants are not only claimed by the pharmaceutical industry, but also the food and cosmetic industry. Herewith, detection and quantification of AOx is of great importance.

Given the increased interest in AOx detection from various resources (foods, supplements, plants) various methods can be employed. Most analytical methods focus on in vitro determination of the antioxidant capacity (AC) following a competitive or noncompetitive reaction, which can be correlated with the anodic area of cyclic voltammetries and/or the electrochemical index using electrochemical measurements [16,17]. In the case of competitive reactions, a competing target molecule is required to compete with the AOx for the reactive species. A good example for this mechanism is the classical technique of chemiluminescence, where the reaction of ROS with chemiluminescence reagents results in a species in an excited state, capable of emitting light. When AOx are added to the solution, and they react with the initiating reactive species, light emission will be inhibited [16]. In the case of non-competitive reactions, AOx compounds interact directly with ROS. Other classical techniques which offer a complex chemical composition analysis of various AOx-containing compounds include chromatographic methods, such as high-performance liquid chromatography used as such [18] or coupled with a 2,2-diphenyl-1-picrylhydrazyl (DPPH) free radical reaction system [19]. Spectroscopic methods are also widely used. Raman spectroscopy allows a fast quality check [20], while Fourier transform infrared spectroscopy (FT-IR) allows the determination of both phenolic content and AC for some rice varieties [21]. Such classical techniques are elaborate, time-consuming, require specific chemical reagents or solvents, and mostly need specialized personnel. To overcome these shortcomings, electroanalytical methods based on sensors and biosensors have been attracting increasing attention and were proven to be more rapid (real-time analysis). Both can be successfully incorporated with the “lab on a chip” technique, offering flexibility, sensitivity and specificity towards the analysed compounds.

The importance of biosensors keeps increasing, since they allow the integration of innovative materials to increase their performance in terms of sensitivity and specificity. The sensitivity of a biosensor depends on the type of transducer (electrochemical, optical) and the technique used to immobilize or functionalize various nanomaterials, polymers that amplify the output signal. Selectivity and specificity depend on the choice of used materials and specific recognition elements such as enzymes or DNA [22].

Although most biosensor configurations focus on the evaluation of the total antioxidant capacity (TAC), this review focuses on biosensors which discriminate among antioxidant species, classes of phenolic compounds, flavonoids or even specific antioxidants such as rutin. Most described biosensors are used for analysis in food or health-related products, but are not limited to these industries. Most reported biosensors are electrochemical ones (amperometric and voltametric), but colorimetric or luminescent assays and optical biosensors are also taken into consideration. A few (bio)electronic tongues are also presented. The described biosensors focus on both direct detection of certain AOx, but also on indirect detection, measuring the induced damage on DNA-based biosensors. Last, a few sensor configurations are also worth mentioning due to their innovation and analytical performances. What highlights the ability of a biosensor to discriminate among phenolic content, AC or a specific AOx compounds lies mainly on the specific behaviour of the (bio)materials (e.g., enzymes, nanomaterials) used for the biosensor architecture. Additionally, the different electrochemical mechanisms allow the identification of each electroactive compound in complex natural samples [16,22]. Enzymes such as Laccase or Tyrozinase are known to favor the detection of phenolic content. Herewith, Tyrozinase oxidizes monophenols and o-diphenols to their corresponding quinone in a two-electron process, whereas Laccase catalyzes the oxidation of several aromatic substrates in a one-electron process. Addition of nanoparticles such as gold nanoparticles, enhance the enzymatic catalytic activity towards specific compounds such as catechol. Cyclodextrins (a family of oligosaccarides) also act as molecular receptors for phenolic substances. The overall antioxidant capacity can be assessed using the principle of inflicted oxidative damage upon DNA or proteins. Thus, the addition of a specific antioxidant or a mixture of antioxidant compounds (including phenolic compounds) in the presence of ROS, will decrease the effect of oxidative damage, allowing the quantification of AC.

## 2. Classification of Antioxidant Species

**Figure 1:**
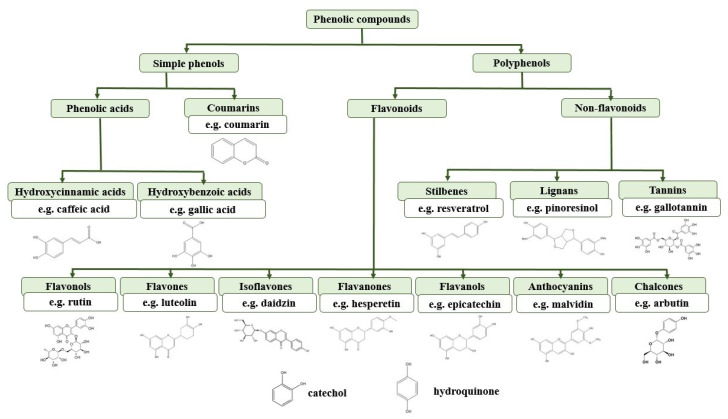
Classification and schematic representation of phenolic compounds.

Several criteria, such as activity, solubility, size, kinetics and occurrence, are used to classify antioxidants [23]. The classification and schematic representation of phenolic compounds is shown in Figure 1.

Most papers in the literature focus on the detection of polyphenols or phenolic compounds due to their health benefits. Polyphenols are usually naturally occurring compounds, found in various plants, and contain multiple functionalities [24]. There are also synthetic phenolic compounds like butylated hydroxyl anisole (BHA), butylated hydroxyltoluene (BHT), and tertiary butyl hydroquinone (TBHQ), for whose detection special enzymatic biosensors have been developed [25]. The activity of polyphenols has been shown to prevent cardiovascular diseases, but also has important anti-inflammatory and anti-viral properties [15]. Polyphenols, alongside vitamins and carotenoids, represent the main classes of dietary AOx. Several classes of polyphenolic compounds can be found in fruits like grapes, apple, pear, cherries and berries [26], or in herbs like lavender [27] and tea [28].

Flavonoids, a widely explored class of polyphenols, are mainly responsible of the colour pigmentation of flowers, fruits and leaves [29]. High contents of flavonols can be found in grapes [30] or walnuts [31]. Vitamins also interrupt free radical chain reactions, and vitamin C (vit C) can be found in various plants [14] and is often consumed as a dietary supplement. There are quite a few ascorbate biosensors to be found in literature, but also vitamins such as vitamins E and D are of interest. A very interesting aspect of biosensors based on ascorbate oxidase is the fact that they enable discrimination between phenols and vit C contribution to the AC of the analysed compound. A review [32] reports a few papers by the group of P.A. Serra in which they highlight the advantages of ascorbate-based biosensors in terms of oxidizing the substrate (vit C) before it reaches the electrode surface. A comparison was performed with the sensor without the enzyme, and the difference in response was used to calculate the vit C selectivity index, thereby underlining vit C detection in a mixture of phenols. Very often, vitamin C is also used as a standard expression of the AC of other analysed AOx [33]. Biosensors for the detection of antioxidant enzymes are less encountered in literature. These enzymes are usually incorporated in the biosensor architecture in order to detect AOx compounds. One such enzyme is peroxidase extracted from a plant known as ice-cream-bean, which is used for the detection of TBHQ [25].

## 3. Biosensors for the Assessment of Phenol Classes

Most biosensors reported in the literature focus on the detection of TAC, where this review focuses on biosensors or even sensors that discriminate among antioxidant compounds or classes of antioxidant compounds. Of great interest is the detection of total phenolic content (TPC), but also the singular or simultaneous detection of certain antioxidants. We present the recent advances achieved in biosensor technologies for AOx evaluation, directed toward phenolic compounds, and the analytical parameters of each biosensor architecture and their corresponding performances are presented in Table 1.

### 3.1. Biosensors for Phenolic Content Assessment

The biosensors for phenolic compounds mainly incorporate two enzymes: tyrosinase (Tyr) and/or laccase (Lac). A laccase crude extract from *Pycnoporus sanguineus* was mixed with graphite and mineral oil to obtain a laccase-based modified carbon paste biosensor [34]. The biosensor was able to detect the TPC in red fruit extracts. In another configuration, the carbon paste was modified with organofunctionalized silica and the new biosensor was used to estimate the TPC of honey samples through the enzymatic oxidation of phenolic compounds, which after reaching a plateau, were electrochemically reduced at the electrode surface [35]. The corresponding cathodic peak currents were used to express the TPC value in gallic acid equivalent (µg (GAE).g^−1^), which were close to the values obtained using the classical assay of Folin-Ciocalteu (FC).

Another simple biosensor architecture was reported by Rodríguez-Sevilla et al. [36]. They immobilized mushroom tyrosinase (Tyr) onto screen-printed electrodes (SPE) using three different techniques: entrapment with water-soluble polyvinyl alcohol (PVA), and crosslinking with glutaraldehyde (GA) in the absence and presence of human serum albumin (HSA). All biosensor configurations were tested in the presence of catechol (CAT), and the best performances were obtained for SPE/Tyr/GA with a sensitivity (S) of 26 ± 4 nA µM^−1^. Finally, this biosensor was used for the quantification of the Trolox Equivalent Antioxidant Capacity (TEAC) of real medicinal plant samples. 

Another simple biosensor design was done by García-Guzmán et al. [37] by modifying a Sonogel-Carbon electrode with Lac from *Trametes versicolor* mixed with GA and Nafion, drop-casting the obtained solution onto the electrode. Phenolic compounds in wines were analysed, first for the detection of individual phenolic compounds (gallic acid; quercetin; rutin; tannic acid; ferulic acid; (+)catechin; (-)epicatechin (ECAT); tyrosol; caffeic acid (CA); vanillic acid; syringic acid; p-coumaric acid and 4-methyl catechol) and second, the total polyphenols content. From the first assay, the authors found that not all selected polyphenols gave an amperometric response; whereas they found good sensitivities for most of the o-diphenols. For the second assay, an increase in the signal compared with the signal obtained for individual phenols was explained either by the synergic effect among the polyphenols or by the contribution from several not individually detectable polyphenols. The same group improved the biosensor a few years later by adding a polymer film, poly(3,4-ethylenedioxythiophene (PEDOT), into the biosensor architecture and using Lac instead of Tyr [38]. Another simple approach based on a carbon paste electrode on which Tyr is immobilized in a Nafion film was presented by Sýs et al. [39]. A mushroom Tyr was used to catalyze the oxidation of p-hydroquinone (HQ) and a satisfactory LoD of 1.6 µM was obtained. An interference study with vitamin C concluded that the vitC:HQ molar concentration ratio should not exceed 1 in order to avoid an interference. For substrate specificity experiments, catechol, resorcinol, phenol and Trolox were used, and the authors concluded that Tyr catalyzes predominantly the oxidation of polyphenols having their hydroxyl group in ortho position. Compounds with this group in meta or para positions need longer time of oxidation, thereby allowing the biosensor to be used for TEAC determinations in wine samples. Another simple and interesting concept was based on α-cyclodextrin modified CPE electrochemical biosensor to detect trans-resveratrol in grape with a sensitivity of S = 310.78 nA μg/L and a limit of detection of LoD = 12 μg/L [40]. The cyclodextrins act as molecular receptors for resveratrol due to their stable “host–guest inclusions” with phenolic substances.

Recent advances in nanomaterial technology have been employed for a variety of biosensors incorporating carbon-based nanomaterials, metallic nanoparticles, or polymers. A solid-contact potentiometric biosensor with two-layer transducer was reported by [41]. The first layer contains a blend of poly(vinyl) chloride carboxylate (PVC-COOH), graphite and potassium permanganate, and the second one contains a mixture of PVC-COOH and graphite, which was deposited on top of the first one. On the last layer, tyrosinase enzyme, extracted from green yellow banana peel, was immobilized by reaction with N-(3-dimethylaminopropyl)-N′-ethylcarbodiimide hydrochloride. Catechol was used as reference phenol, and its concentration was plotted against a relative potential, obtained through the difference between initial and final potential. In the same manner, honey and propolis samples were analysed for their phenolic content, and the results were expressed in mg of GAE by 100 g of sample. For most samples, they found very similar values when compared to the classical Folin–Ciocalteu method. Additionally, using catechol as reference, Cabaj et al. immobilized Tyr in an electrochemically synthesized copolymer based on N-nonylcarbazole derivatives [42]. A platinum electrode was coated with a thin polymer film through electropolymerization, while fungal Tyr from *Agaricus Bisporus* was electrodeposited. A temperature study for Tyr electrodeposition was conducted, and the authors found that T = 47 °C is optimal. They recorded the current response in PBS buffer (pH 7.0) containing 0.2 mM catechol for the temperature range (0–50 °C), and they assigned this high thermal stability to the porosity of the electrosynthesized polymer 2,7-BSeC (poly [2,7-bis(selenophene)-N-nonylcarbazole]), which reduces the conformational flexibility and prevents enzyme unfolding. Both cathechol and L-Dopa were analysed as phenolic compounds, and the sensitivity for catechol was determined to be 2.45 µA mM-1, where LoD was found to be quite low (0.02 µM) compared to other biosensor configurations at that time.

The Langmuir–Blodgett technique was used to develop films containing mushroom tyrosinase, lutetium bisphthalocyanine and arachidic acid (Tyr/AA/LuPc2) adsorbed onto ITO glass [43] and was used for the detection of several phenol derivatives. The main advantage was that the Langmuir–Blodgett film could be cycled up to 50 times for which the percentage of decay was lower than 4%. 

Nanoparticles are widely used to either improve the enzymatic catalytic activity, enhance biosensor selectivity or gain more control over the electrode microenvironment [13,16,44]. Carbon-based nanomaterials such as single- or multi-walled carbon nanotubes (SW-, MW-CNT), carbon nanofibers are available on a large scale and are most often incorporated into biosensor architectures. De Oliveira et al. [25] used MWCNT mixed with graphite powder and sepiolite clay mineral containing peroxidase. The homogeneous paste, incorporated into a plastic syringe, was used as a working electrode for the detection of the synthetic TBHQ. Square wave voltammetry (SWV) was used to monitor the reduction potential of TBHQ at the biosensor surface. The LoD and quantification limit (LoQ) were determined to be 0.41 and 1.25 mg L^−1^. Furthermore, the biosensor was used to determine TBHQ recovery from salad dressing samples, and the results were comparable with those obtained with the classical HPLC technique. Also, the biosensor was sufficiently sensitive to be used in the quality control of foods. MWCNTs alongside reduced graphene oxide (GO) were used by Vlamidis et al. [45] for the detection of polyphenols in fruit juices. Several biosensor architectures were tested and compared, using both laccase from *Trametes versicolor* and tyrosinase from mushroom, immobilized onto GCE, employing different agents such as bovine serum albumin (BSA) and glutaraldehyde as crosslinking agents, chitosan, and Nafion. The Lac-based biosensor showed a better performance towards catechol detection and the biosensor configuration was optimized as follows: GCE was modified with a mixture of reduced GO and MWCNT (denoted as “hybrid” layer), followed by Lac immobilization. To extend the lifetime of the biosensor, several protective biomembranes were tested, and a mixture of 20 mg mL^−1^ BSA and 2.5% (v/v) GA, denoted BSA-GA1 was found to be optimal. Finally, the chronoamperometric response of the biosensor was recorded at 0 V and the calibration graphs were constructed by plotting the reduction current as a function of catechol concentration. This is the first work using Lac for catechol detection in this review, obtaining a LoD of 0.3 µM, while in the case of Tyr, the LoD is slightly higher (0.5 µM). Only the biosensor Pt/2,7-BSeC/Tyr [42] managed to obtain a lower detection limit for catechol using Tyr. The GCE/hybrid/Lac/BSA-GA1 biosensors performance was tested towards a variety of polyphenols (gallic acid, pyrogallol, 2,3-di-hydroxybenzoic acid, dopamine, epicatechin, catechin, rutin, caffeic acid and chlorogenic acid) displaying a good sensitivity towards most compounds. For real sample analysis, fruit juices were analysed comparing the results obtained with Lac- and Tyr-based biosensors for the total polyphenolic content.

The group of Zgardzińska [46] used microporous carbon fibers as electro-conductive immobilization matrixes for Lac. The authors prepared two different types of carbon fibers (CFs) by cellulose carbonization. The differences were mainly in the specific surface area and their preparation process. Both CFs (CFA and CFB) had delignified cellulose as precursor, but for CFB, a ZnCl_2_ activator was used, flattening the microfibers. Laccase from *Trametes versicolor* was coupled with CFs-modified graphite-rod electrodes, both showing a very similar response during CVs. The optimal working parameters were determined for ABTS (2,2′- Azino-bis(3-ethylbenzthiazoline-6-sulfonic acid)) and catechol. For both compounds, the amperometric detection is based on the reduction of oxidized electroactive products (ABTS cation radicals and semiquinone intermediates) formed during the enzymatic reaction. The detection limit to catechol for both biosensors was less than 5 μM, where the sensitivity for CFA was 1000 A M^−1^ m^−2^ and for CFB 1137 A M^−1^ m^−2^. The CFA-modified biosensor was used for analysis of catechol in raw and spiked (0.25 mM CAT) wastewater samples and the results were compared with those obtained with HPLC. Between the two techniques, a significant difference could be noticed and was attributed to the fact that wastewater could have contained some catechol-like components that were also oxidized by the enzyme, due to the broad specificity of laccase. When using the “standard addition mode” on the CFA-modified biosensor, its sensitivity towards raw wastewater was very similar to that of catechol standard solution (5.54 ± 0.29 mA mM^−1^ and 5.6 ± 0.4 mA mM^−1^), demonstrating the high accuracy of biosensing.

Gold nanoparticles (AuNPs) are also widely used due to their enhanced catalytic properties. A layer-by-layer (LbL) biosensor architecture containing AuNPs was developed by Salvo-Comino et al. [47]. Using an ITO electrode, successive immersions of the substrate into electrocatalytic solutions, containing chitosan (CHI), positively charged, and two negatively charged solutions with copper(II) phthalo-cyanine-tetrasulfonic acid tetrasodium salt (CuPcS) and AuNPs were performed. Two LbL architectures were tested, [(CHI)-(AuNP)-(CHI)-(CuPcS)]_2_ and [(CHI)-(CuPcS)-(CHI)-(AuNP)]_2_, and on each multilayer covered electrode, either Tyr (from *Agaricus bisporus*) or Lac (from *Trametes versicolor*) was immobilized. The LbL structure with higher roughness and pore size (meaning the presence of (CuPcS) as top layer) was shown to facilitate the diffusion of catechol, and among the two enzymes, Tyr showed the best catalytic properties, obtaining the lowest LoD to date, at 0.85 nM. The use of two enzymes was to better evaluate the electron mediator capability of the LbL, knowing that Tyr oxidizes monophenols and o-diphenols to the corresponding quinone (in a two-electron process), whereas Lac catalyzes the one-electron oxidation of several aromatic substrates. Chronoamperograms were recorded for increasing concentrations of catechol, and a sensitivity of 0.681 A M^−1^ was calculated for the best performing LbL/Tyr configuration. No real sample analysis was done in this work. Another biosensor configuration containing AuNPs alongside graphene nanoplatelets was developed by Zrinski et al. [48]. Graphene nanoplatelet-modified screen-printed carbon electrodes were fabricated by printing with a graphene nanoplatelets modified carbon ink on a ceramic substrate. Next, a solution of AuNPs was drop-casted on the surface, followed by the enzyme solution containing a mixture of nafion, ethanol, water and Lac from *Rhus vernicifera*. After drying, the electrode was used for HQ detection. The electrochemical behaviour of HQ was also evaluated at each modification step, and in the end, the TEAC assay was chosen as the reference method. Great attention was given to the sensor modification, parameter optimization and characterization. The biosensor analytical performances are presented in Table 1, and for repeatability and reproducibility, an RSD (relative standard deviation) of ± 2% and ± 3% was obtained. The authors also made a comparison of their biosensor performances with literature, and where lower LoDs were found, the linear range was shorter. The wider linear range reported in this work was considered suitable for easy evaluation of TEAC in real samples. First, several representative phenolic antioxidants and related compounds were analysed using hydrodynamic amperometry, and the results were presented in a histogram as equimolar ratio of HQ. There was low or no interference with paracetamol, dopamine, ascorbic acid, where a ratio up to 41.2% was found for phenols such as CA, phenol, p-coumaric acid or syringic acid. Using the same method, the total phenolic antioxidant capacity was assessed as Trolox equivalent and hydroquinone equivalent, and the results were compared with those obtained from the TEAC assay. The values obtained with the newly developed Lac biosensor were comparable with those from the conventional spectrophotometric method.

**Figure 2:**
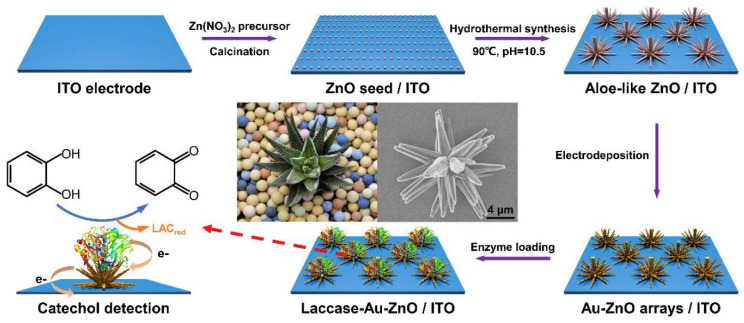
Schematic illustration of the preparation of aloe-like Au–ZnO arrays on an ITO electrode (reproduced from [49] with permission of Elsevier).

Due to the increased catalytic properties of gold, Liu et al. [49] developed aloe-like Au–ZnO micro/nanoarrays for the detection of catechol. A lot of effort was put into the growth of the aloe-like Au–ZnO arrays on an ITO electrode, and this process is illustrated in Figure 2. Zinc oxide (ZnO) was chosen since it is a well-known semiconducting material on which enzymes can easily adsorb. ZnO/ITO was prepared through a modified wet chemical process, after which the aloe-like ZnO was coated with adsorbed Zn^2+^; followed by the electrodeposition of gold from a HAuCl_4_ electrolyte solution.

Due to electrostatic force, AuCl^4-^ can easily accumulate on the surface of the ZnO. The nanostructure evolution of the arrays was continuously optimized, and the growth mechanism of the crystals was closely monitored in order to obtain the best configuration. The electrochemical behaviour of the Au-ZnO-based electrodes was monitored using electrochemical impedance spectroscopy (EIS), which showed that the introduction of AuNPs efficiently improved the conductivity of ZnO, benefiting of a current signal amplification. After this step, the enzyme was immobilized, and the biosensor performances were tested in the presence of catechol. Since phenol, HQ and resorcinol (RS) may produce interference, the authors tested the influence of these phenolic compounds on the oxidation of catechol. Using CV, only the addition of CAT generated a clearly defined redox signal when compared to the other compounds. Using amperometry in different pH electrolytes, both HQ and CAT generated a current response from the oxidation process at pH 8.25. This happened due to the over-oxidation behaviour of the ZnO material in alkaline environments. However, for the neutral pH of 7.0, only the current change caused by CAT had a significant intensity, while phenol, HQ and RS produced little interference. Herewith, for increasing CAT concentrations, a sensitivity of 131 µA mM^−1^ and a LoD of 25 nM were calculated. The biosensor showed good reproducibility with an RSD of 3.29%, and showed an exceptional stability in the first 10 days, retaining most of its initial sensitivity, starting to decrease up to 15% after 30 days. The biosensor was also tested in real water samples and the results were compared with those of HPLC. No CAT was detected in the water samples, so manual addition was required, and the recovery varied between 98.90% and 101.10%. All results prove that a very sensitive, selective biosensor was developed, and its use in real samples is reliable.

**Figure 3:**
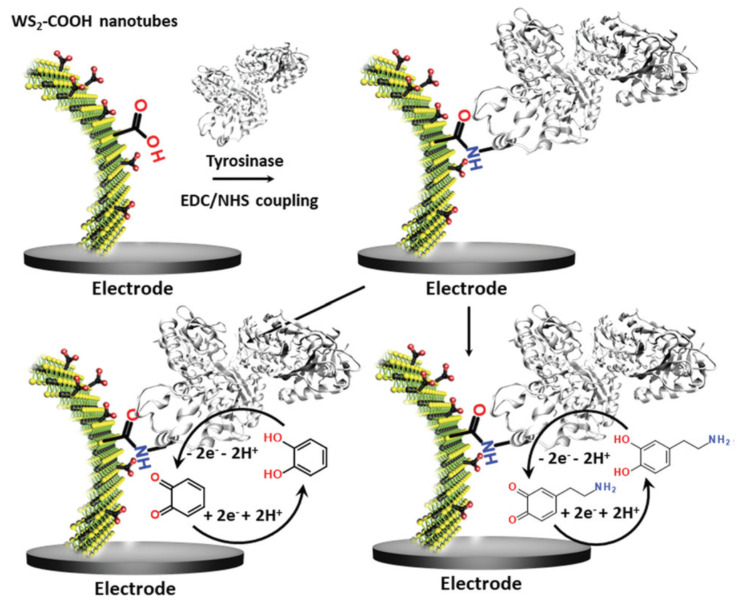
Sketch of the functionalization of WS_2_ modified glassy carbon electrodes with the enzyme tyrosinase via a standard EDC/NHS coupling reaction. These modified bioelectrodes served in the detection of catechol (bottom left) and dopamine (bottom right) at −0.2 V vs. Ag^+^/Ag. (reproduced from [50] with permission of RSC Publishing).

A very interesting and less encountered nanoparticle type was used and functionalized by Palomar et al. [50]. The authors used tungsten disulfide nanotubes (WS_2_) functionalized with carboxylic acid functions (WS_2_-COOH). The carboxylic acid groups served as an anchor for the immobilization of Tyr from mushroom via a standard EDC/NHS coupling reaction. The electrochemical behaviour of the WS_2_-COOH films on GCEs was monitored using CV in organic media, where the irreversible oxidation of WS_2_ is shown. The WS_2_-COOH nanotube films with a controlled thickness of 6.2 µm, were then used as a support for the immobilization of tyrosinase, as shown in Figure 3.

For this, the electrode was first incubated with PBS (phosphate buffered saline), pH 7.4, containing EDC (N-(3-Dimethylaminopropyl)-N0-ethylcarbodiimide hydrochloride), NHS (N-hydroxysuccinimide) and DMAP (4-(dimethylamino)pyridine) for 12 h, followed by another immersion of 12 h in Tyr solution. The authors used the newly developed biosensor for the detection of catechol and dopamine due to the enzymatic oxidation of the phenol ring, present in both compounds. Chronoamperometric measurements were done at −0.2 V vs. SCE, and the current density was proportional to the CAT concentration. CAT will be regenerated during the reduction at the electrode, allowing the amplification of the signal, leading to an increased sensitivity of 152.5 mA L cm^−2^ mol^−1^. Although the sensitivity is quite low compared to other reports, the linear range is similar. This can be explained by the presence of the functionalized WS_2_ nanotubes, which allow a high amount of enzyme immobilization. The low sensitivity on the other hand was explained either due to rapid oxygen consumption and/or reduced permeability of the WS_2_-COOH film, as well as lack of conductivity at the used potential. In that same manner as CAT, dopamine was also detected with a sensitivity of 6.2 mA L cm^−2^ mol^−1^ over a linear range between 0.5 and 10 µM. The authors did not obtain spectacular results, but they showed that the WS_2_-COOH nanotubes can be promising components for the development of electrochemical biosensors.

### 3.2. DNA- and Protein-Based Biosensors for Phenolic Content Assessment

**Figure 4:**
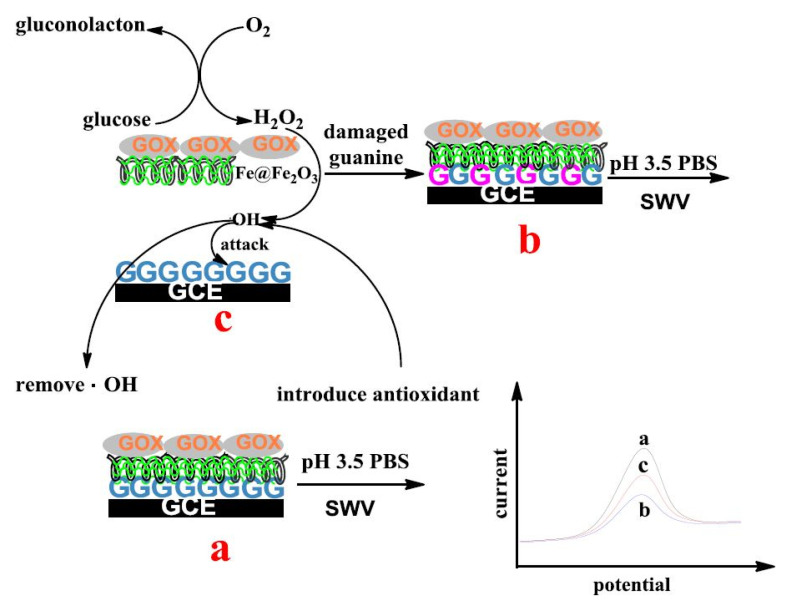
Mechanism of the biosensor and the detection method. SWV (square wave voltammetry). Reproduced from [52] with permission from ESG publisher under a Creative Commons Attribution 4.0 International License http://creativecommons.org/licenses/by/4.0/).

DNA-based biosensors use DNA as a recognition element, and their performance is assessed based on the principle of oxidative damage inflicted on the DNA by ROS/RNS. By adding an antioxidant to the solution, the oxidative damage should decrease, indirectly evaluating the antioxidant capacity of the analysed phenolic compounds [51]. Either single- or double-stranded DNA can be immobilized on the electrode surface, or one of the amino acids can be used. Wang et al. [52] chose guanine to be added in a composite membrane, as shown in Figure 4. 

The membrane was built on a GCE by first immersing the electrode into a buffered guanine solution, which adsorbed on the activated GCE surface at a potential of +0.4 V for 180 s under constant stirring. Then, the guanine-modified surface was sequentially covered with Fe@Fe_2_O_3_ and glucose oxidase (GOX). The resulting biosensor was named GOX/PDDA-Fe@Fe_2_O_3_/G/GCE. The biosensor was built in such way that the reactions between GOX, glucose and Fe@Fe_2_O_3_ generated a hydroxyl radical, which oxidized the guanine. SWV was used to determine the biosensor performances, first in PBS, pH 3.5, recording the peak current as i_0_; and second, in PBS, pH 5.0, containing 50 mmol L^−1^ glucose in the absence or presence of an antioxidant, after incubation for a certain time. The peak current was recorded as i_t_, and the guanine oxidative damage, as well as AC, was detected by calculating the signal change for G. Vit C was used as a well-known radical scavenger in order to show the effect of incubation time on the intensity of the peak potential Δi_p_ (in the presence and absence of vit C). In the presence of vit C, Δi_p_ was smaller, indicating some protective effects on the oxidation of G. In the same manner, several phenolic compounds were tested, and their AC was expressed as AOT% = i_t_/i_0_ × 100. The AOT% was determined for CA, coumaric acid and resveratrol, where the highest value was obtained for resveratrol and the lowest for CA. The authors highlight the fact that the G damage process and the protection of phenolic compounds was achieved via a series of biochemical reactions in the composite membrane, simulating the in vivo processes.

Although most DNA-based biosensors found in the literature focus on DNA damage and the detection of TAC, we found two examples where DNA was used as a biopolymer. The first was the work of Mello et al. [53], which describes the use of a DNA additive in the architecture of an enzymatic biosensor, and the focus is on the relationship between total antioxidant activity (TAA) and total phenol content of *Ilex paraguariensis* extracts. This is the only work we found that does not monitor the oxidative damage on DNA, but works as a classical electrochemical biosensor for the detection of chlorogenic acid (CGA). The CPE was prepared by immobilizing the DNA additive and horseradish peroxidase solution (HRP) on silica-titanium with GA. After drying, graphite powder and mineral oil were added to the mixture in order to obtain a homogeneous paste, which was put into a glass tube. The biosensor response was measured as the difference between total and residual current using amperometry. The development and optimization parameters of the biosensor were described in a previous work [54]. The TAA was evaluated by the DPPH colorimetric UV–Vis assay, on the basis of IC_50_, which represents the AOx concentration needed to reduce 50% of the initial amount of DPPH˙. A low IC_50_ value indicates the presence of strong AOx compound in the extracts. By plotting the results obtained for TAA against polyphenols compound concentration using the biosensor method, a linear relationship was observed with correlation coefficients > 0.9 for all analysed samples. Thereby, the authors highlight that the determination of the TPC was representative in terms of AOx compounds, and interferents like ascorbate or carbohydrates showed no response; thus, the phenol content is the main source of AOx in *Ilex paraguariensis* extracts. Another work using DNA as a biopolymer was authored by Ferreira Garcia et al. [55], where the effect of different biosensor modifiers was evaluated on a laccase CPE. The laccase crude extract was obtained through submerged fermentation of *Pycnoporus sanguineus* in a specific growth medium. First, the Lac crude extract was mixed with graphite powder (CPL), to which various modifiers (GA, BSA, chitosan, DNA, silica, titanium dioxide, activated and non-activated CNTs) were added, left to dry, and finally mixed with mineral oil. The homogeneous paste was then filled into an electrode support. After the optimization of parameters and the voltametric characterization of all electrode configurations, the best results were obtained by CPL-DNA:CNT. The presence of activated CNTs significantly improved the biosensor sensitivity, which was further improved by the presence of DNA, whose biocompatibility lead to a better enzymatic activity of the immobilized enzyme. A calibration plot for the detection of rutin was constructed, obtaining an LoD of 12 µM and an LoQ of 38 µM. To further optimize the CPL-DNA:CNT biosensor for the detection of phenolic compounds, the influence of conditioning time and starting potential were evaluated and determined to be optimal at a conditioning time of 30 s and starting potential of 0.5 V. These parameters were kept for an affinity assay; where the relative response of CPL-DNA:CNT against equimolar concentrations of different phenolic compounds (CAT, gallic acid, rutin, CA, CGA, phenol and chlorophenol) was monitored. The highest response was obtained for CAT, closely followed by gallic acid and rutin. For real sample analysis, the total phenolic content in crude coffee samples was determined and compared with the classical Folin–Ciocalteu assay. The total phenolic values for both methods were expressed as GAE mg mL^−1^, and they were found to be in good agreement, with an RSD% of the biosensor lower than the one obtained for the FC method.

**Table 1:** Partial applications of various biosensor architectures for the detection of phenolic antioxidants.

Biosensors	Index	Sample	Detection Technique	Linear Range	LoD	Ref
Lac-CPE ^1^	TPC	red fruits	DPV	-	-	[34]
Lac-CPE	TPC	honey	DPV	-	-	[35]
SPE ^2^/Tyr/GA ^3^	catechol	medicinal plants	amperometry	0 ≤ [CAT] ≤ 136 µM	1.5 ± 0.6 µM	[36]
Lac/SNGC ^4^	gallic acid	wine	amperometry	-	0.011 mg L^−1^	[37]
PEDOT ^5^-Tyr/SNGC	caffeic acid	beer, wine	amperometry	10 ≤ [CA] ≤ 300 µM	4.33 µM	[38]
CPE/Tyr/Nafion	hydroquinone	red wine	amperometry	20 ≤ [HQ] ≤ 120 µM	1.6 µM	[39]
^6^ α-CD-CPE	trans-resveratrol	grape extracts	DPV	30 ≤ [resv] ≤1000 µg L^−1^	12 μg L^−1^	[40]
Transducer -Tyr	catechol	honey, propolis	potentiometry	9.3 × 10^−7^ ≤ [CAT] ≤ 8.3×10^−2^ M	7.3 × 10^−7^ M	[41]
Pt ^7^/2,7-BSeC ^8^/Tyr	catechol	-	DPV	1.5 ≤ [CAT] ≤ 80 µM	0.02 µM	[42]
Tyr/AA ^9^/LuPc_2_^10^	caffeic acid	-	CV	10 ≤ [CA] ≤ 400 µM	1.98 µM	[43]
CPE–CNT ^11^–SEP ^12^–nafion–peroxidase	TBHQ	salad dressing samples	SWV	1.65 ≤ [TBHQ] ≤ 9.82 mg L^−1^	0.41 mg L^−1^	[25]
GCE ^13^/hybrid/Lac/BSA ^14^-GA1	catechol	fruit juices	amperometry	1 ≤ [CAT] ≤ 300 µM	0.3 µM	[45]
CFA ^15^-CFB ^16^/Lac	catechol	wastewater	amperometry	-	< 5 µM	[46]
[(CHI ^17^)-(AuNP ^18^)-(CHI)-(CuPcS ^19^)]_2_-Tyr	catechol	-	amperometry	2.4 ≤ [CAT] ≤ 20 µM	8.55 × 10^−4^ µM	[47]
LACC/AuNP/GNPl ^20^/SPCE ^21^	hydroquinone	wine, blueberry syrup	hydrodynamic amperometry	4 ≤ [HQ] ≤ 130 µM	1.5 µM	[48]
Lac/Au–ZnO ^22^/ITO ^23^	catechol	environmental water	amperometry	0.075 ≤ [CAT] ≤ 1100 µM	25 nM	[49]
GC ^24^/WS_2_-COOH ^25^/tyrosinase	catechol	-	amperometry	0.6 ≤ [CAT] ≤ 70 µM	-	[50]
CPL ^26^-DNA ^27^:CNT	rutin	coffee	DPV	-	12 µM	[55]
protein-based solid biosensor with Cu(II)-Nc ^28^ assay	epicatechin	herbal infusions	absorbance	12.5≤ [ECAT] ≤ 150 µM	1.2 µM	[56]
protein-based solid biosensor with Fe(II)-Fz ^29^ assay	epicatechin	herbal infusions	absorbance	25≤ [ECAT] ≤ 250 µM	0.5 µM	[57]

^1^ Carbon paste electrode; ^2^ Screen-printed electrode; ^3^ Glutaraldehyde; ^4^ Sonogel carbon electrode; ^5^ Poly(3,4-ethylenedioxythiophene; ^6^ α-cyclodextrin; ^7^ Platinum; ^8^ Poly[2,7-bis(selenophene)-N-nonylcarbazole]; ^9^ Arachidic acid; ^10^ Lutetium bisphtalocyanine; ^11^ Carbon nanotubes; ^12^ Sepiolite; ^13^ Glassy carbon electrode; ^14^ Bovine serum albumin; ^15^ Carbon fibre A; ^16^ Carbon fibre B; ^17^ Chitosan; ^18^ Gold nanoparticle; ^19^ Copper(II) phthalo-cyanine-tetrasulfonic acid tetrasodium salt; ^20^ Graphene nanoplatelets; ^21^ Screen printed carbon electrode; ^22^ Gold-zinc oxide; ^23^ Indium tin oxide; ^24^ Glassy carbon; ^25^ Tungsten disulphide nanotubes with carboxylic acid functions; ^26^ Carbon paste laccase; ^27^ Deoxyribonucleic acid; ^28^ Copper(II)- neocuproine; ^29^ Iron(II)-ferrozine.

Returning to the principle of oxidative damage detection, protein-based sensors are an alternative to DNA-based biosensors. In the group of R. Apak, Akyüz et al. published two papers on protein-based solid biosensors, evaluating protein damage. The first biosensor was used for the determination of Cu(II)-induced pro-oxidant activity of several phenolic compounds alongside non-phenolic vitamin C [56]. The protein-based solid sensor was prepared by completely separating the egg white from the yolk, to which water and CaCl_2_ was added dropwise as protein precipitation agent. The Ca−proteinate precipitate was filtered, washed, dried and grinded, after which the dried protein residue was ready to use. The powder was diluted in phosphate buffer, pH 7.4, copper(II) solution and various concentrations of standard antioxidant solutions or herbal plant infusions. The final solution was vortexed, centrifuged, and the upper liquid decanted. The remaining protein-based sensor was washed and solutions of neocuproine (Nc), NH_4_Ac buffer and water were added. The solution was again agitated, filtered and the absorbance at 450 nm was recorded against a reagent blank. The blank was missing the antioxidant standard. Such an assay involves the reduction of Cu(II) ions to Cu(I) due to the presence of the antioxidant compounds, where Cu(I) binds to the solid biosensor. The protein-bound Cu(I), is an indicator of the pro-oxidant activity of antioxidants on proteins, which can be colorimetrically determined through absorbance measurements at 450 nm with Nc. Phenolic compounds from several classes were chosen for analysis: phenolic acids (gallic acid and CGA), flavanols (CAT and ECAT) and flavonols (quercetin—QUE and myricetin—MYR). The pro-oxidant activity of these compounds was generally visible above the critical concentration of 2.50 µM. ECAT was chosen as a standard compound, and analytical performances such as LoD (1.2 µM) and LoQ (4.0 µM) were determined using two methods, the biosensor and the carbonyl assay (considered a standard method as comparison for pro-oxidative status detection), where the biosensor assay was shown to be more sensitive. Vit C had the least pro-oxidant activity, while CAT and ECAT had similar effects and QUE showed the highest activity. For statistical comparison of the two methods, the total pro-oxidant activities of ECAT standard and sage extract were calculated as ECAT equivalent. The obtained pro-oxidant activities can be easily converted to antioxidant activity (given as the ratio between the slopes of the calibration plot for the test compound and reference compound); however, these values were not calculated by the authors. The recovery values were close to 100%, demonstrating that the solid biosensor used with the Cu(II)-Nc assay was suitable to determine the pro-oxidant activity of plant extracts containing polyphenolic compounds. The authors also highlight a close relationship between the pro-oxidative behaviour and the ion reducing ability of transition metals of phenolic compounds. In their second work [57], the group uses the same solid biosensor for developing a novel Fe(III)-ferrozine (Fz) spectrophotometric pro-oxidant activity assay. The Fe(III) reducing ability generated by AOx compounds is an indirect measure of their pro-oxidant activity, since reduced ions may generate ROS. The formed Fe(II) will bind to the solid biosensor, enabling the measurement of the absorbance at 562 nm of the Fe(II)-ferrozine chelate. The solutions were prepared in the same manner, only the buffer was adjusted to pH 5.5, and Nc was replaced with Fz. Again, ECAT was chosen as the standard compound and analytical performances such as LoD (0.5 µM) and LoQ (1.8 µM) were determined. Compared to previous Cu(II)-Nc assay, the performances were improved with ~58%. The pro-oxidant activity of several phenolic compounds was determined, followed by ECAT recoveries from herbal infusions. Although a very elaborated assay as compared to what a biosensor stands for, quite low values for the detection limits of ECAT were obtained.

### 3.3. Biosensor Trends and Perspectives

This chapter refers to new concepts, trends and promising perspectives, where a classical “biosensor” will not always be described. In response to the demand for fast, sensitive and selective techniques, bioelectronic tongues (BioETs) have been designed, combining achievements of chemometric analysis with the unique properties of biological compounds. Such a device is described by Medina-Plaza et al. [58], based on a previously presented work [43]. Based on the results obtained using principal component analysis (PCA), which demonstrated that a multisensor system was able to discriminate phenols according to the number of phenolic groups present in the structure, the authors formed an array of three electrodes in order to discriminate among phenolic antioxidants in the food industry. The three (bio)sensors were developed as follows: Langmuir–Blodgett (LB) films were prepared using a PBS-NaCl subphase, onto which a mixture of arachidic acid and lutetium bisphtalocyanine was spread. Twenty such monolayers were deposited onto an ITO glass surface, obtaining the first sensor (AA/LuPc_2_). For the other two enzymatic biosensors, onto 10 monolayers of AA/LuPc_2_, 10 monolayers of enzyme/AA/LuPc_2_ were added. Two enzymes were used, Lac form *Trametes versicolor* and Tyr from mushroom. After preparation, the Enz/AA/LuPc_2_ films were treated with glutaraldehyde to covalently immobilize the enzyme. Cyclic voltammetry was used to evaluate the response of the three-electrode array towards six phenolic compounds: a monophenol (vanillic acid- VA), two ortho-diphenols (CAT and CA), one para-diphenol (HQ) and two triphenols (gallic acid and pyrogallol). The responses, calculated from the peaks associated with AOx, were highly reproducible, lower than 1.5%. The reproducibility of different sensors containing the same enzyme was also found to be lower than 1.75%. The AA/LuPc_2_ film showed a quasi-reversible and intense redox pair, associated with the presence of LuPc_2_, which acts as a mediator, amplifying the signal. It can be also noticed, that the LuPc_2_ redox pair appears at different positions with varying intensities, specific to each analysed compound. The authors also found that the presence of the enzyme increased even more the peak intensities associated with the phtalocyanine ring (~ −0.2 V). From the presented voltammograms, they also concluded that they attained an important degree of cross-selectivity due to the array of sensors. The authors observed significant differences in the response of the Enz/AA/LuPc_2_ electrodes, attributed to the enzyme specificity, on which a nice discussion was elaborated. The detection limits were determined for each biosensor by measuring the associated peak intensities towards increasing concentrations of the phenolic compounds. The peak intensity linearly increased with the AOx concentration, and the lowest LoD was calculated for gallic acid with the Lac/AA/LuPc_2_ biosensor, with a value of 4.1 × 10^−8^ mol L^−1^. Overall, the detection limits for Enz/AA/LuPc_2_ were at least one order of magnitude lower than for AA/LuPc_2_. In the case of CAT, for both Enz/AA/LuPc_2_ biosensors the LoDs were calculated to be around ~ 4.7 x 10^−7^ mol L^−1^, values that are among the lowest (Table 1). Since the array of voltametric electrodes generated signals of intrinsic complexity and cross-selectivity, the authors were able to discriminate the phenolic compounds using PCA. The obtained PCA score plot showed separated clusters (three clusters corresponding to the mono-, di- and tri-phenols). To validate the results obtained with PCA, the BioET was used in musts prepared from grapes of different varieties. The voltammograms clearly showed the response of the phenolic groups present in must, and the peak intensities and positions were related with the total polyphenol index measured by standard chemical methods, which depends on the grape variety. Applying PCA, the biosensor array was able to discriminate grapes according to the grape variety, and the results were validated with the chemical composition of the grape juice.

**Figure 5:**
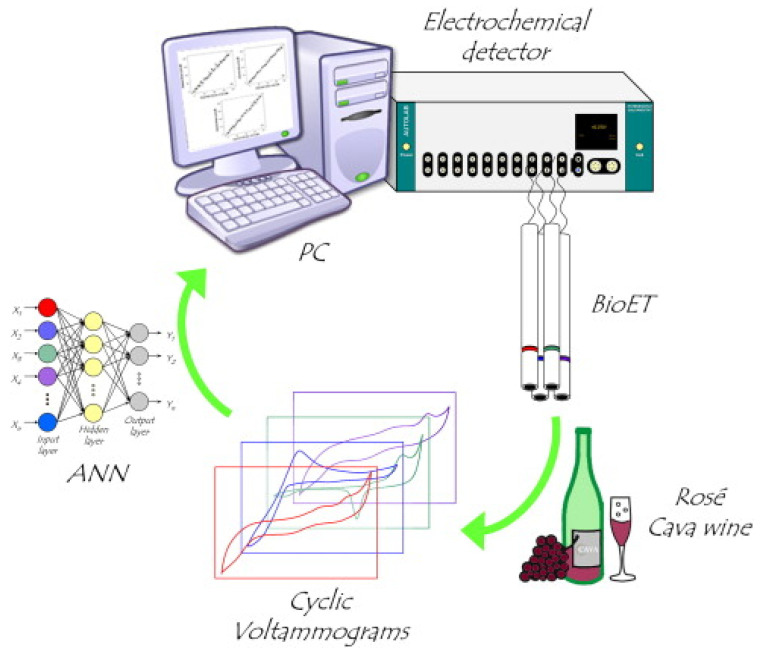
Schematic representation of the BioET approach (reproduced from [59] with permission of Elsevier Publishing).

Another voltametric BioET was described by Ceto et al. [59], comprising an array of four enzyme-modified (bio)sensors. The electrodes were prepared by mixing resin with its corresponding hardener in a ratio of 20:3 (w/w), then adding 15% (w/w) graphite and 2% (w/w) modifier (either Tyr, Lac or copper NPs); after which the mixture was homogenized and left to dry. One electrode was kept as a blank, without any modifier. The electrochemical cell was formed by the four (bio)sensors as a working electrode array, with a double junction Ag/AgCl reference and a platinum counter electrode. The obtained data were further processed with various chemometric tools, including PCA. Since the (bio)sensor array had been already successfully used in the resolution of phenolic compound mixtures in wine samples, the authors proceeded to directly analyse 20 varieties of wine, by recording a complete voltammogram with each sensor for each sample. The working principle of the BioET is presented in a schematic approach in Figure 5. 

Overall, the oxidation of the phenolic compounds was noticed in all cases, while the reducing behaviour occurred only in some of the samples. Obtaining a large amount of data with variability among them and among the different biosensors, the condition for developing an ET is fulfilled. Since the BioET array clearly recognizes phenolic content, PCA was used to detect any similarities between the obtained voltametric responses. Thereby, the PCA plot shows how the samples group according to their phenolic content. It could be clearly observed that one cluster groups samples with low phenolic content, while another cluster groups the samples with the highest phenolic content, obtaining six clusters in total. No specification was made on any specific phenolic compound, all the data being regarded as the total phenolic index. To validate the BioET response, modelling software was used (ANN), about which we will only specify that the theoretical expected values were very close to the predicted ones.

An interesting topic worth debating is the replacement of biocomponents with NPs or polymers capable of mimicking the biological component in question. Such systems gain more and more attention due to increased stability and long-term use. Cui et al. [60] found a promising alternative for natural enzymes in a catalytically active nanomaterial by synthesizing a porphyrin-based porous organic polymer, named FePPOP-1. Porphyrin-based porous organic polymers (PPOPs) present a large specific surface area, tuneable pore structures and high stability, properties which facilitate electron and mass transfer. Introducing metalloporphyrins in a POPs framework, the PPOPs skeleton itself will act as a catalyst, integrating the polymeric structure into the family of nanozymes. FePPOP-1 was obtained by the reaction between iron 5,10,15,20-tetrakis-(4′-bromophenyl) porphyrin and 1,3,5-triethynylbenzene, presenting a high peroxidase activity and low LoDs, as will be shown. To verify the peroxidase-like activity of FePPOP-1, the authors used the typical peroxidase substrate of 3,3′,5,5′-Tetramethylbenzidine (TMB) in the presence of H_2_O_2_, where FePPOP-1 catalyses the oxidation of TMB. The decomposition of H_2_O_2_ into ˙OH radicals was highlighted by using terephthalic acid as a fluorescent probe. The authors presented the following reaction mechanism: FePPOP-1 catalyses the decomposition of H_2_O_2_ into ˙OH, which oxidizes the TMB substrate. By varying the concentration of FePPOP-1, the fluorescence intensity gradually increased, proving H_2_O_2_ decomposition. The detection of H_2_O_2_ was achieved by adding different concentrations into a mixture of acetate buffer (pH 3.8), TMB and FePPOP-1, after which UV–Vis spectra were recorded. Since the catalytic activity of FePPOP-1 depends on H_2_O_2_ concentration, a linear dependence of the TMB absorbance with increasing H_2_O_2_ concentration was observed, and an LoD of 6.5 µM was determined. H_2_O_2_ is also considered a free radical, which means that in the presence of an antioxidant species, H_2_O_2_ scavenging should occur. Thereby, the antioxidant capacity of the phenolic compounds gallic acid and tannic acid (TA), alongside vit C was achieved through indirect detection. First, it should be mentioned that in the AOx-free FePPOP-1-TMB mixture, TMB oxidation resulted in a blue colour, which faded upon AOx addition. Thereby, UV–Vis absorption allows the colorimetric detection of AOx. To highlight this process, the AOx were mixed with acetate buffer, TMB and FePPOP-1. Next, a fixed concentration of 50 mM H_2_O_2_ was added to the mixture for 3 min and the UV–Vis spectra were recorded. The absorbance intensity decreased with increasing AOx concentration. After construction, the calibration plots for each compound, vit C, gallic acid and TA, could be detected to be as low as 0.35, 0.75 and 0.048 μM. The antioxidative abilities of the compounds were evaluated in the following order: TA > vit C > gallic acid. Table 1 presents only one work [37] that uses gallic acid as a standard, and if we compare the two LoDs, this assay is more sensitive compared to the amperometric assay.

The concept of indirect detection of AOx capacity has been widely discussed in the literature, and although it is not the main topic in this review, in addition to the previous reference, we found another interesting biosensor architecture based on the indirect detection of gallic acid via H_2_O_2_ inhibition. An amperometric biosensor using Prussian Blue (PB) and xanthine oxidase (XOD) was developed by Becker et al. [61]. SPE were first modified with PB, which acts as a suitable mediator for H_2_O_2_ reduction. Next, a homogeneous mixture containing XOD solution and Azide-unit Pendant Water-soluble Photopolymer (PVA/AWP) was spread on the SPE. The electrode was then exposed to neon light at +4 °C to allow polymerization. Amperometric measurements were performed in a dark glass cell in K-PBS at a working potential of -0.1 V vs. Ag/AgCl. After signal stabilization, 5 mM hypoxanthine (HX—the specific substrate for XOD) was successively added. This procedure was repeated in the absence and presence of different AOx samples, and the corresponding calibration plots were constructed. The evaluation of the AOx capacity is based on monitoring the H_2_O_2_ produced during the oxidation of HX to uric acid in the presence of the enzyme. The generated H_2_O_2_ is then reduced at the polarized surface of the biosensor. The addition of AOx reduces the H_2_O_2_ concentration. The biosensor was used for the determination of gallic acid antioxidant capacity, by recording the amperometric response as a function of successive addition of 5 mM HX in the absence and presence of several gallic acid concentrations ranging between 12.5 and 200 µM. With increasing AOx concentration, the current signal decreased and its AC correspondent was expressed in percentage. For a concentration of 200 µM, an AC of 42% was calculated. The authors presented further analytical data on the biosensor performance for HX detection in the absence and presence of 12.5 µM gallic acid as follows: the biosensor response was linear only in the absence of gallic acid in the range of 1.0–75 µM HX, the LoDs were 2.17 and 0.72 µM in the absence and presence of gallic acid, and the LoQ also decreased from 7.15 to 2.42 µM in the presence of gallic acid. The biosensor was then successfully applied in real samples of Amazonian fruits.

## 4. Conclusions

Although a large number of publications focus on the evaluation of TAC, using direct or indirect (ROS scavenging) methods, we managed to bring together several publications focusing on the specific detection of a phenolic compound, with a final chapter dedicated to concepts and methodologies worth mentioning due to their novelty, complexity or improved performance. Most described biosensors were also applied in real sample analysis, mainly related to food or health industries. The majority of biosensors were electrochemical, using mostly amperometric, followed by voltametric (DPV) techniques to evaluate their performances. The newest trends are based on the synthesis of novel materials or combination of various nanomaterials due to their physical, chemical or optical properties. The authors also look to replace biological compounds with materials which are perfectly capable of mimicking the biological activity (e.g., nanozymes).

Even though the performance of the biosensors is continuously improving, their miniaturization and portability is still an issue that needs to be overcome. Since most of the described biosensors have applicability in the food industry, their portability should be an important aspect. 

A rigorous comparison and analysis of all presented biosensors is difficult to obtain due to the many differences and variables determined by each research group. Even though many biosensors use laccase, the enzyme is extracted from various sources, introducing already a factor of uncertainty. Catechol is one of the main phenolic standards used in seven assays, and the LoD varies from 8.55 × 10^−4^ to 5 µM. A difference of 4 orders of magnitude is quite significant, though different enzymes, NPs, polymers and detection techniques were employed.

Biosensors are therefore a fast, reliable alternative to classical techniques. Depending on the scope of research, however, biosensors can be used as a complementary technique alongside classical ones, especially when the focus of research is based on understanding the role of each molecule in a matrix. Plant matrices are very complex, and as some reviewed articles highlighted, there are scenarios where biosensors detect a class of phenols or TPC, depending on their architecture. On the other hand, focusing on specific analytes, biosensors have the advantage to lower the detection limit and increase selectivity and specificity using various nanomaterials or polymers. The versatility of (bio)sensors is of great importance, since they can be used independent or combined with standard classical methods depending on the scope of research.

## References

[1] Dasgupta A., Klein K. (2014). Antioxidants in Food, Vitamins and Supplements.

[2] Badarinath A.V., Rao K.M., Madhusudhana C.C., Ramkanth S., Rajan T.V.S., Gnanaprakash K. (2010). A Review on In-vitro Antioxidant Methods: Comparisions, Correlations and Considerations. Int. J. Pharmtech Res..

[3] Lobo V., Patil A., Phatak A., Chandra N. (2010). Free radicals, antioxidants and functional foods: Impact on human health. Pharmacogn. Rev..

[4] Senoner T., Dichtl W. (2019). Oxidative Stress in Cardiovascular Diseases: Still a Therapeutic Target?. Nutrients.

[5] Asmat U., Abad K., Ismail K. (2015). Diabetes mellitus and oxidative stress-A concise review. Saudi Pharm. J..

[6] Gilgun-Sherki Y., Melamed E., Offen D. (2001). Oxidative stress induced-neurodegenerative diseases: The need for antioxidants that penetrate the blood brain barrier. Neuropharmacology.

[7] Pohanka M. (2014). Alzheimer´s disease and oxidative stress: A review. Curr. Med. Chem..

[8] Zuo T., Zhu M., Xu W. (2015). Roles of Oxidative Stress in Polycystic Ovary Syndrome and Cancers. Oxidative Med. Cell. Longev..

[9] Cairns R.A., Harris I.S., Mak T.W. (2011). Regulation of cancer cell metabolism. Nat. Rev. Cancer.

[10] Nimse S., Pal D. (2015). Free radicals, natural antioxidants, and their reaction mechanisms. RSC Adv..

[11] Prior R.L., Wu X., Schaich K. (2005). Standardized Methods for the Determination of Antioxidant Capacity and Phenolics in Foods and Dietary Supplements. J. Agric. Food Chem..

[12] Buratti S. (2008). A low-cost and low-tech electrochemical flow system for the evaluation of total phenolic content and antioxidant power of tea infusions. Talanta.

[13] David M., Şerban A., Popa C., Florescu M. (2019). A Nanoparticle-Based Label-Free Sensor for Screening the Relative Antioxidant Capacity of Hydrosoluble Plant Extracts. Sensors.

[14] Suryakumar G., Gupta A. (2011). Medicinal and therapeutic potential of Sea buckthorn (Hippophae rhamnoides L.). J. Ethnopharmacol..

[15] Oroian M., Escriche I. (2015). Antioxidants: Characterization, natural sources, extraction and analysis. Food Res. Int..

[16] David M., Serban A., Radulescu C., Danet A.F., Florescu M. (2019). Bioelectrochemical evaluation of plant extracts and gold nanozyme-based sensors for total antioxidant capacity determination. Bioelectrochemistry.

[17] Bunaciu A.A., Danet A.F., Fleschin S., Aboul-Enein H.Y. (2015). Recent Applications for in Vitro Antioxidant Activity Assay. Crit. Rev. Anal. Chem..

[18] Wantusiak P.M., Glód B.K. (2012). Application of UV detection in HPLC in the total antioxidant potential assay. Open Chem..

[19] Zhang Y., Li Q., Xing H., Lu X., Zhao L., Qu K., Bi K. (2013). Evaluation of antioxidant activity of ten compounds in different tea samples by means of an on-line HPLC–DPPH assay. Food Res. Int..

[20] Maia L.F., Ferreira G.R., Costa R.C.C., De Lucas N.C., Teixeira R.I., Fleury B.G., Edwards H.G.M., De Oliveira L.F.C. (2014). Raman Spectroscopic Study of Antioxidant Pigments from Cup Corals Tubastraea spp.. J. Phys. Chem. A.

[21] Jantasee A., Thumanu K., Muangsan N., Leeanansaksiri W., Maensiri D. (2013). Fourier Transform Infrared Spectroscopy for Antioxidant Capacity Determination in Colored Glutinous Rice. Food Anal. Methods.

[22] Ye Y., Ji J., Sun Z., Shen P., Sun X. (2020). Recent advances in electrochemical biosensors for antioxidant analysis in foodstuff. TrAC Trends Anal. Chem..

[23] Aziz M.A., Diab A.S., Mohammed A.A. (2019). Antioxidant Categories and Mode of Action. Antioxidants.

[24] Handique J., Baruah J.B. (2002). Polyphenolic compounds: An overview. React. Funct. Polym..

[25] De Oliveira T.R., Grawe G.F., Moccelini S.K., Terezo A.J., Castilho M. (2014). Enzymatic biosensors based on ingá-cipó peroxidase immobilised on sepiolite for TBHQ quantification. Analyst.

[26] Pandey K.B., Rizvi S.I. (2009). Plant polyphenols as dietary antioxidants in human health and disease. Oxidative Med. Cell. Longev..

[27] Baptista R., Madureira A.M., Jorge R., Adão R., Duarte A., Duarte N., Lopes M.M., Teixeira G. (2015). Antioxidant and Antimycotic Activities of Two Native Lavandula Species from Portugal. Evid. Based Complement. Altern. Med..

[28] Almajano M.-P., Carbo R., Jiménez J.A.L., Gordon M. (2008). Antioxidant and antimicrobial activities of tea infusions. Food Chem..

[29] Gil E.D.S., Couto R.O.D. (2013). Flavonoid electrochemistry: A review on the electroanalytical applications. Rev. Bras. de Farm..

[30] Masa A., Vilanova M., Pomar F. (2007). Varietal differences among the flavonoid profiles of white grape cultivars studied by high-performance liquid chromatography. J. Chromatogr. A.

[31] Pereira J.A., Oliveira I., Sousa A., Valentão P., Andrade P.B., Ferreira I., Ferreres F., Bento A.A., Seabra R., Estevinho L.M. (2007). Walnut (Juglans regia L.) leaves: Phenolic compounds, antibacterial activity and antioxidant potential of different cultivars. Food Chem. Toxicol..

[32] Torrinha Á., Amorim C.G., Montenegro M.C.B.S.M., Araujo A.N. (2018). Biosensing based on pencil graphite electrodes. Talanta.

[33] Tienaho J., Sarjala T., Franzén R., Karp M. (2015). Method with high-throughput screening potential for antioxidative substances using Escherichia coli biosensor katG′::lux. J. Microbiol. Methods.

[34] De Macêdo I.Y.L., Garcia L.F., Neto J.R.D.O., Leite K.C.D.S., Ferreira V.S., Ghedini P.C., Gil E.D.S. (2017). Electroanalytical tools for antioxidant evaluation of red fruits dry extracts. Food Chem..

[35] Neto J.R.D.O., Rezende S.G., Lobón G.S., Garcia T.A., De Macêdo I.Y.L., Garcia L.F., Alves V.F., Torres I.M.S., Santiago M.F., Schimidt F. (2017). Electroanalysis and laccase-based biosensor on the determination of phenolic content and antioxidant power of honey samples. Food Chem..

[36] Rodríguez-Sevilla E., Ramírez-Silva M.-T., Romero-Romo M., Ibarra-Escutia P., Palomar-Pardavé M. (2014). Electrochemical Quantification of the Antioxidant Capacity of Medicinal Plants Using Biosensors. Sensors.

[37] García-Guzmán J.J., Hernández-Artiga M.P., De León L.P.-P., Bellido-Milla D. (2015). Selective methods for polyphenols and sulphur dioxide determination in wines. Food Chem..

[38] García-Guzmán J.J., López-Iglesias D., Cubillana-Aguilera L., Lete C., Lupu S., Palacios-Santander J.M., Bellido-Milla D. (2018). Assessment of the Polyphenol Indices and Antioxidant Capacity for Beers and Wines Using a Tyrosinase-Based Biosensor Prepared by Sinusoidal Current Method. Sensors.

[39] Sýs M., Pekec B., Kalcher K., Vytřas K. (2013). Amperometric Enzyme Carbon Paste-Based Biosensor for Quantification of Hydroquinone and Polyphenolic Antioxidant Capacity. Int. J. Electrochem. Sci..

[40] Pekec B., Oberreiter A., Hauser S., Kalcher K., Ortner A. (2012). Electrochemical Sensor Based on a Cyclodextrin Modified Carbon Paste Electrode for Trans -Resveratrol Analysis. Int. J. Electrochem. Sci..

[41] Draghi P.F., Fernandes J.C.B. (2017). Label-free potentiometric biosensor based on solid-contact for determination of total phenols in honey and propolis. Talanta.

[42] Cabaj J., Jędrychowska A., Swist A., Sołoducho J. (2016). Tyrosinase Biosensor for Antioxidants Based on Semiconducting Polymer Support. Electroanalysis.

[43] Apetrei C., Alessio P., Constantino C., De Saja J., Rodriguez-Mendez M.L., Pavinatto F., Fernandes E.G.R., Zucolotto V., Oliveira O. (2011). Biomimetic biosensor based on lipidic layers containing tyrosinase and lutetium bisphthalocyanine for the detection of antioxidants. Biosens. Bioelectron..

[44] Dhand C., Dwivedi N., Ying A.N.J., Lakshminarayanan R., Ramakrishna S., Loh X.J., Verma N.K., Beuerman R.W. (2015). Methods and strategies for the synthesis of diverse nanoparticles and their applications: A comprehensive overview. RSC Adv..

[45] Vlamidis Y., Gualandi I., Tonelli D. (2017). Amperometric biosensors based on reduced GO and MWCNTs composite for polyphenols detection in fruit juices. J. Electroanal. Chem..

[46] Kavetskyy T., Smutok O., Demkiv O., MaŤko I., Švajdlenková H., Šauša O., Novák I., Berek D., Čechová K., Pecz M. (2020). Microporous carbon fibers as electroconductive immobilization matrixes: Effect of their structure on operational parameters of laccase-based amperometric biosensor. Mater. Sci. Eng. C.

[47] Salvo-Comino C., González-Gil A., Rodriguez-Valentin J., García-Hernandez C., Martín-Pedrosa F., Garcia-Cabezon C., Rodriguez-Mendez M.L. (2020). Biosensors Platform Based on Chitosan/AuNPs/Phthalocyanine Composite Films for the Electrochemical Detection of Catechol. The Role of the Surface Structure. Sensors.

[48] Zrinski I., Pungjunun K., Martinez S., Zavašnik J., Stanković D., Kalcher K., Mehmeti E. (2020). Evaluation of phenolic antioxidant capacity in beverages based on laccase immobilized on screen-printed carbon electrode modified with graphene nanoplatelets and gold nanoparticles. Microchem. J..

[49] Liu T., Zhao Q., Xie Y., Jiang D., Chu Z., Jin W. (2020). In situ fabrication of aloe-like Au–ZnO micro/nanoarrays for ultrasensitive biosensing of catechol. Biosens. Bioelectron..

[50] Palomar Q., Gondran C., Lellouche J.-P., Cosnier S., Holzinger M. (2020). Functionalized tungsten disulfide nanotubes for dopamine and catechol detection in a tyrosinase-based amperometric biosensor design. J. Mater. Chem. B.

[51] Diculescu V., Chiorcea-Paquim A., Brett C.M. (2016). Applications of a DNA-electrochemical biosensor. TrAC Trends Anal. Chem..

[52] Wang X., Chen R., Sun L., Yu Z. (2014). Study on the Antioxidant Capacities of Four Antioxidants Based on Oxidizing Guanine in a Composite Membrane. Int. J. Electrochem. Sci..

[53] Mello L.D., Kubota L.T. (2014). Antioxidant capacity of Ilex paraguariensis extracts by using HRP-based biosensor. Lat. Am. Appl. Res..

[54] Mello L.D., Sotomayor M.D.P.T., Kubota L.T. (2003). HRP-based amperometric biosensor for the polyphenols determination in vegetables extract. Sens. Actuators B Chem..

[55] Garcia L.F., Benjamin S.R., Marreto R.N., Lopes F.M. (2015). Laccase Carbon Paste Based Biosensors for Antioxidant Capacity. The Effect of Different Modifiers. Int. J. Electrochem. Sci..

[56] Akyüz E., Başkan K.S., Tütem E., Apak R. (2017). Novel Protein-Based Solid-Biosensor for Determining Pro-oxidant Activity of Phenolic Compounds. J. Agric. Food Chem..

[57] Akyüz E., Başkan K.S., Tütem E., Apak R. (2020). Novel Iron(III)−Induced Prooxidant Activity Measurement Using a Solid Protein Sensor in Comparison with a Copper(II)−Induced Assay. Anal. Lett..

[58] Medina-Plaza C., De Saja J., Rodriguez-Mendez M.L. (2014). Bioelectronic tongue based on lipidic nanostructured layers containing phenol oxidases and lutetium bisphthalocyanine for the analysis of grapes. Biosens. Bioelectron..

[59] Cetó X., Capdevila J., Mínguez S., Del Valle M. (2014). Voltammetric BioElectronic Tongue for the analysis of phenolic compounds in rosé cava wines. Food Res. Int..

[60] Cui C., Wang Q., Liu Q., Deng X., Liu T., Li D., Zhang X. (2018). Porphyrin-based porous organic framework: An efficient and stable peroxidase-mimicking nanozyme for detection of H2O2 and evaluation of antioxidant. Sens. Actuators B Chem..

[61] Becker M.M., Ribeiro E.B., Marques P.R.B.D.O., Marty J.-L., Nunes G.S., Catanante G. (2019). Development of a highly sensitive xanthine oxidase-based biosensor for the determination of antioxidant capacity in Amazonian fruit samples. Talanta.

